# The Impact of Frailty on Adverse Outcomes in Geriatric Hip Fracture Patients: A Systematic Review and Meta-Analysis

**DOI:** 10.3389/fpubh.2022.890652

**Published:** 2022-06-30

**Authors:** Yanhong Song, Ziyi Wu, Huihui Huo, Ping Zhao

**Affiliations:** ^1^Department of Anesthesiology, Shengjing Hospital of China Medical University, Shenyang, China; ^2^Department of Pediatrics, Shengjing Hospital of China Medical University, Shenyang, China

**Keywords:** hip fracture, frailty, adverse outcomes, meta-analysis, elders

## Abstract

**Objective:**

With an aging population and advances in medicine, more research focuses on health and longevity in geriatric adults. Recently, frailty has gradually emerged to assess physical conditions. Frailty can be generally described as a multi-dimensional situation of increased vulnerabilities to both endogenous and exogenous stressors. The objective of the review was to evaluate the predictive value of frailty on adverse outcomes in geriatric hip fracture patients.

**Materials and Methods:**

We searched PubMed, Embase, Web of Science, and the Cochrane library for relevant literature about the connection between frailty and poor outcomes in hip fracture elders.

**Results:**

Eleven studies involving a total of 45,979 participants were selected in our study. Our results indicated that frailty could significantly predict postoperative and in-patient complications (OR, 1.46; 95% CI, 1.13–1.90; *I*^2^ = 77.4%). Frail elders had higher risk of inpatient mortality (OR, 1.68; 95% CI, 1.26–2.25; *I*^2^ = 0.0%), 6-month mortality (OR, 1.46; 95% CI, 1.25–1.72; *I*^2^ = 0.0%) and ≥1-year mortality (OR, 2.24; 95% CI, 1.66–3.04; *I*^2^ = 91.3%). Furthermore, the risk of prolonged hospital stays was 1.15 times more likely in frail patients (95% CI, 1.03–1.28; *I*^2^ = 14.8%).

**Conclusion:**

Frailty can predict adverse outcomes effectively in geriatric hip fracture patients.

**Systematic Review Registration:**

https://www.crd.york.ac.uk/PROSPERO/#recordDetails.

## Introduction

With social development and medical advances, the average life expectancy is increasing significantly, which leads to an accelerating population aging process. It is predicted that approximately 17% of the global population is expected to be over 65 years old by 2050 (1). The growing demand for surgery among older patients is driven by a combination of increased life expectancy and improved surgical safety (2). The bone mass and muscle mass of the organism start to decrease significantly from approximately fifty years of age, and they can interact with each other through paracrine and endocrine signals (3). Osteoporosis, muscle atrophy, and many other reasons have put the older persons at high risk for fractures (4, 5). Hip fractures have become a worldwide public health threat, with data indicating that the 1-year mortality rate after hip fracture surgery may exceed 20% (6). It is estimated that by 2050, the population of hip fractures is predicted to reach 4.5 million worldwide, and nearly 30% will occur in China (7). Due to the unsatisfactory quality of life and inevitable complications associated with conservative treatment, surgery is now mostly preferred when the physical condition of the patient is compatible with the surgical indications. Risk stratification of geriatric patients appears to be necessary regardless of therapeutic measures.

It has long been widely accepted that advancing age is an increasing risk contributor to undesirable postoperative outcomes. However, the emerging perspective holds that frailty (age-related impairment of physical reserves and resilience) seems to be a better predictor of complication occurrence and increased mortality in comparison to age (8, 9). Frailty is characterized by a multi-dimensional and dynamic condition of increased vulnerability and decreased resistance to stress in the older persons as a result of reduced physiological reserves. Current perspectives suggest that a combination of chronic inflammatory responses, nutritional and metabolic imbalances, oxidative stress, and changes in hormone levels are involved in the development of frailty (10–12). Frailty often affects multiple systems, such as the neuromuscular, endocrine, and immune systems (13). In a weakened state, a relatively small stimulus can cause an increased probability of a series of negative clinical events. Frailty assessment can reflect the ability of older adults to regulate the internal environmental stability and resist stress. Therefore, early identification of frailty has become an essential component of health promotion in geriatric aging.

Now-a-days, a modest but growing number of clinical studies have researched the association between frailty and undesirable outcomes in geriatric hip fracture patients. However, these findings are not convincing owing to small samples and inconsistent results, and there has not been a meta-analysis to systematically and quantitatively synthesize the results of multiple independent studies. Hence, we perform the systematic review and meta-analysis to assess the status of frailty assessment in clinical practice. Our objectives were to assess the predictive value of frailty for adverse outcomes among geriatric hip fracture patients (death, complications, adverse discharge destination, and prolonged length of hospital stays).

## Materials and Methods

### Literature Search

This study protocol has been registered on PROSPERO (CRD42022291453). Two researchers independently searched databases, including PubMed, Web of Science, Embase, and the Cochrane library for publications from 1 January 1990, to 31 October 2021. The English search terms included “frail,” “frailty,” and “hip fracture.” We also obtained additional articles by checking references to the original articles to ensure that all available clinical trials were included. Articles were considered for further analysis which reported the frailty assessment application for the patients with hip fracture. The search strategy was summarized in Supplementary File 1.

### Eligibility Criteria

Inclusion criteria: (1) The study population consisted of hip fracture patients with a mean age ≥75 years (including femoral neck fractures, subtrochanteric fractures, and intertrochanteric fractures, (2) Prospective or retrospective cohort studies, (3) application of frailty assessment, and (4) reported the relationship between frailty and poor outcomes including mortality, complications, adverse discharge destination, and prolonged hospitalization.

Two reviewers (YS and ZW) retrieved the literature and independently evaluated the potentially eligible studies. When disagreements arose, we discussed and consulted with the corresponding author (PZ) to reach a consensus.

### Data Extraction and Study Quality

Two investigators, respectively extracted relevant data from the included articles according to standard data tables and documented the study characteristics. The following details were collected: the first author and publication year, type of study design, country, frailty measure, sample, mean age, and outcome characteristics.

The quality evaluation of the included research was conducted by the Newcastle–Ottawa Scale (NOS), which can assess the risk of bias in observational studies in the next main domains: study participant selection, comparability, and outcome measures (14). The scores ranged from 0 to 9 points, and all studies were classified as low quality (<7) and high quality (≥7) following NOS.

### Statistical Analysis

We extracted the ORs and 95%CIs from each included studies and Stata version 17.0 was utilized for the meta-analysis.

The following criteria and methods were used to assess heterogeneity in the trials. (1) *I*^2^ < 50% indicated the presence of homogeneity, and the fixed-effects model was selected for performing the meta-analysis. (2) *I*^2^ ≥ 50% indicated the existence of heterogeneity, and the random-effects model was used to conduct the meta-analysis.

Sensitivity analyses were applied to assess the robustness of results and subgroup analysis was carried out on the factors that may cause heterogeneity. The study design (retrospective vs. prospective cohorts) and the regions for implementation of the experiment were considered to divide the studies into different subgroups.

## Results

### Study Screening

**Figure 1:**
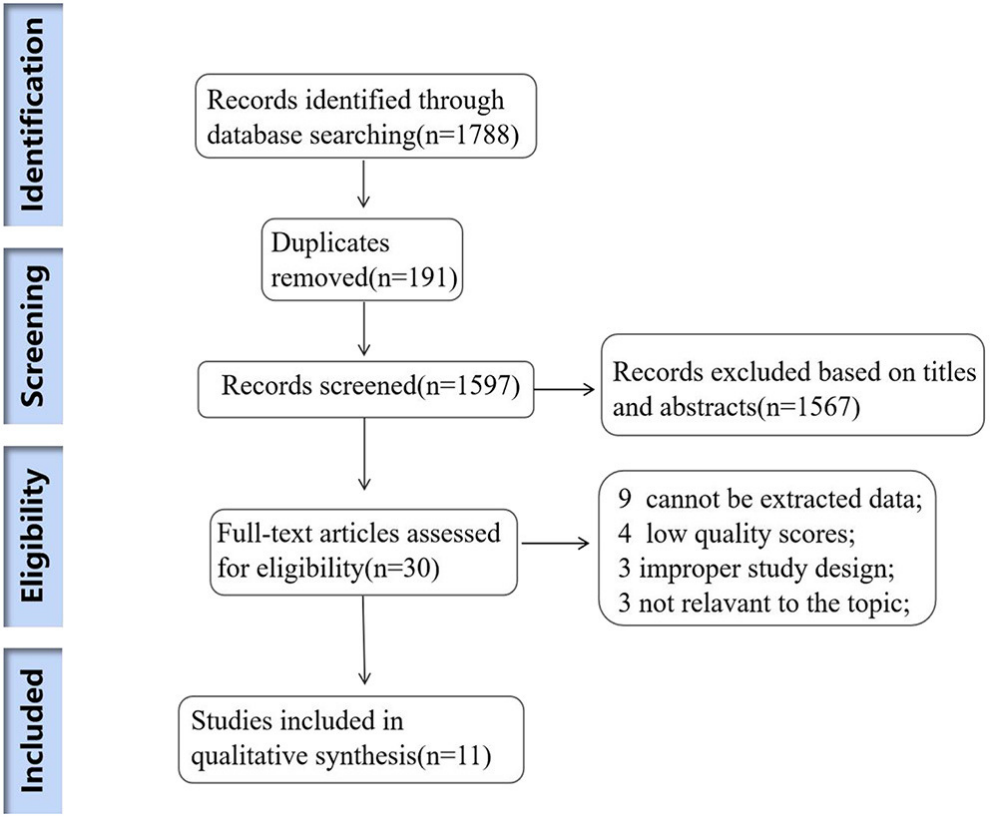
Flow diagram of the study selection process.

We originally retrieved 1,788 studies in total. After removing the duplicates, 1,597 were excluded based on the titles and abstracts. The full texts of the remaining 30 articles were screened, 3 articles were excluded because of the improper study design, 3 articles were irrelevant to our topic and in another 9 studies, ORs and 95% CI were not reported. Finally, only 11 articles were included in the review. The detailed screening process was presented in Figure 1.

### Characteristics and Quality of the Included Studies

The basic characteristics of the 11 studies included were summarized in Table 1. All 11 articles were published between 2014 and 2021. Two reviewers (YS and ZW) independently assessed the quality using the Newcastle–Ottawa Scale (NOS) (More details in Supplementary File 2). In 11 included studies, 4 were prospective cohort studies (6, 16, 18, 19) and the remaining 7 were retrospective cohort studies (15, 17, 20–24). The studies were implemented in the Netherlands (19), America (15), Singapore (16), Canada (17), China (18), Korea (20, 22), Australia (21), Italy (6), Japan (24) and England (23). The average age ranged from 78 to 86.5 years old (More details in Table 1).

**Table 1:** Characteristics of all the studies included in the meta-analysis.

**References**	**Study design**	**Country**	**Frailty Measure**	**Sample**	**Mean age**	**Outcomes**	**Quality assessment (NOS)**
Patel (15)	R	America	19-Item mFI (0–1)	481	81.05 (SD:8.45)	1-year mortality	7
Kua (16)	P	Singapore	MFC derived from the original Fried criteria (0–5); REFS (0–18)	82	79.1 (SD:9.6)	Postoperative complications;6-month mortality	6
Chan (17)	R	Canada	The Canadian Study of Health and Aging CFS 2018 (1–9)	422	82.5 (SD:8.4)	In-patient complications; Adverse discharge destination	7
Chen (18)	P	China	the Chinese-Canadian Study of Health and Aging Clinical Frailty Scale (CSHA-CFS) (1–7)	245	78(range: 53–97)	6-month mortality	8
Winters (19)	P	Netherlands	GFI (0–15) Hospital Safety Management (VMS) frailty score (0–2)	277	83 (SD:6.6)	≥1-year mortality	7
Choi (20)	R	Korea	Hip-MFS (0–14)	481	80.4 (range: 75.3–85.3)	Postoperative complications; 6-month mortality; 1-year mortality; prolonged hospital stay	7
Jorissen (21)	R	Australia	Frailty index (0–0.41)	4,771	86 (range 82–90)	≥1-year mortality	8
Choi (22)	R	Korea	Hip-MFS (0–14)	242	81.5 (SD:6.7)	Postoperative complications; 6-month mortality	7
Thorne (23)	R	England	NHFS; CFS	2,422	85 (interquartile range 78–90)	In-patient mortality; 1-year mortality; prolonged hospital stays	6
Pizzonia (6)	p	Italy	mFI-19	364	86.5(SD: 5.65)	1-year mortality	7
Shimizu (24)	R	Japan	HFRS	36,192	83.6 (SD: 6.7)	In-patient mortality	7

### Meta-Analysis Results

#### Mortality

**Figure 2:**
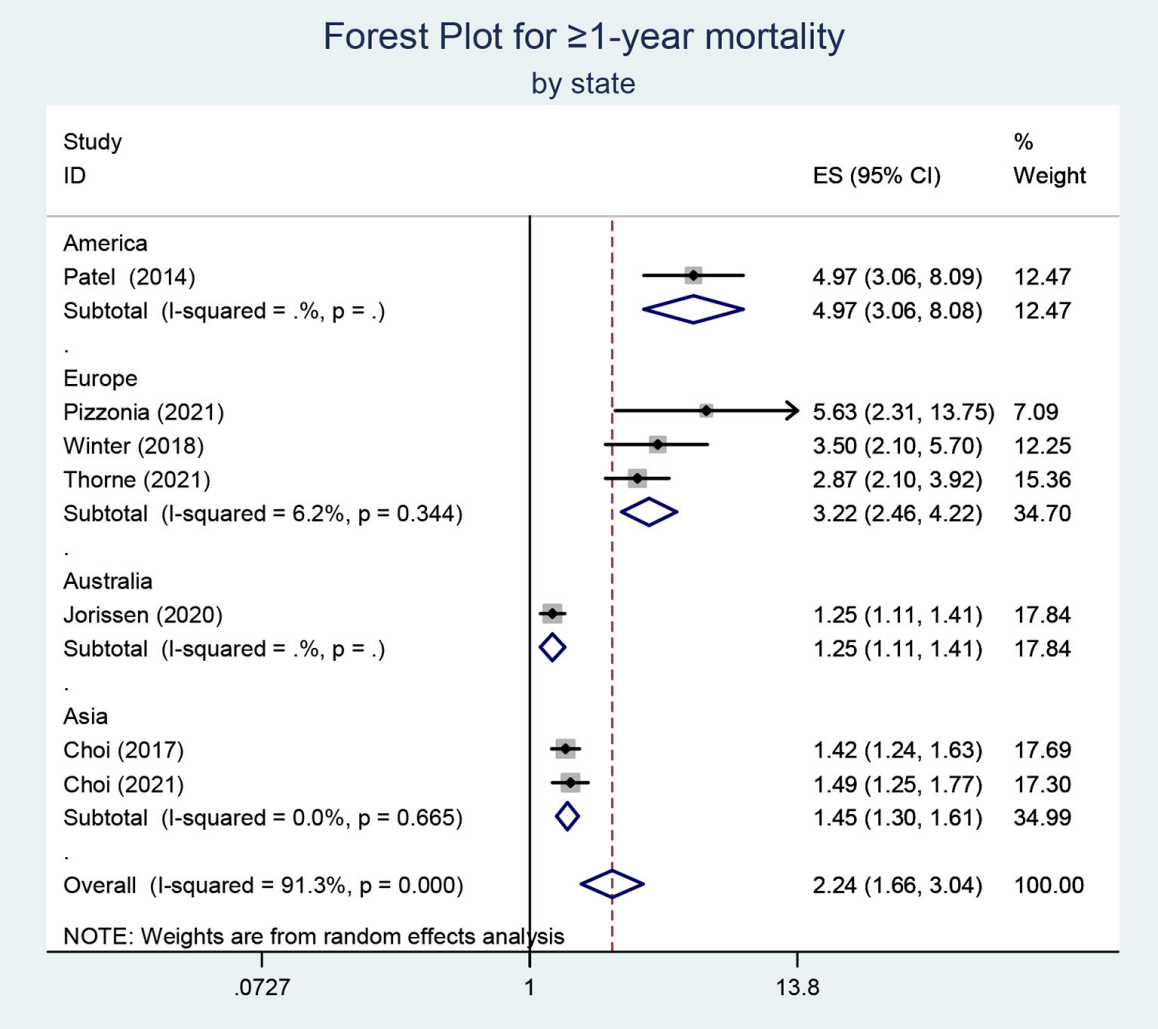
Subgroup analysis for the studies reporting ≥ 1-year mortality.

All the findings of our meta-analysis have been summarized in Table 2. (The forest plots can also be found in Supplementary File 3). Debilitating physical conditions may lead to increased mortality. The effect of frailty on mortality of different durations was explored in our included studies, including in-patient, 6-month, and ≥1-year mortality. For in-patient and 6-month mortality, tests of heterogeneity both suggested that *I*^2^ = 0.0% and a fixed-effects model was applied. The heterogeneity of studies reporting long-term (≥1-year) mortality is quite high (*I*^2^ = 91.3%) and a random-effects model was employed for the meta-analysis. We understand that publication bias is a test that is necessary only when the number of included documents meets the standard, but the quantitative criteria are currently controversial. In our analysis, only 7 articles were included. Egger's regression test was performed and the results indicated the existence of publication bias (*P* < 0.05). In addition, we conducted subgroup analysis according to the state of included studies. The heterogeneity might come from the groups of Europe and America. The results of the European and American studies showed a greater correlation between frailty and long-term mortality (OR, 3.22; 95% CI, 2.46–4.22; OR, 4.97; 95% CI,3.06–8.08, respectively) (Figure 2). The subgroup analysis was also implemented based on the experimental design and the results suggested that the heterogeneity may be derived from retrospective studies. Above all, the results indicated that frailty might be an excellent predictor of increased mortality after hip fracture.

**Table 2:** Results of meta-analysis.

**Study**	**ES**	**95% CI**	**Weight (%)**
**Postoperative/in-hospital complications**			
Kua (16)	3.42	(1.09, 10.75)	4.75
Chan (17)	4.80	(2.10, 10.80)	8.48
Choi (20)	1.24	(1.12, 1.38)	44.38
Choi (22)	1.25	(1.09, 1.43)	42.39
Total (95% CI) *I*^2^ = 63.3%, (*p* <0.0001)	1.46	(1.13, 1.90)	100.00
**In-patient mortality**			
Shimizu (24)	1.57	(1.12, 2.21)	73.73
Thorne (23)	2.03	(1.15, 3.58)	26.27
Total (95% CI) *I*^2^ = 0.0%, (*p* <0.0001)	1.68	(1.26, 2.25)	100.00
**6-month mortality**			
Kua (16)	1.95	(0.19, 20.28)	0.46
Chen (18)	4.60	(1.05, 20.04)	1.16
Choi (22)	1.40	(1.03, 1.92)	25.95
Choi (20)	1.46	(1.21, 1.76)	72.43
Total (95% CI) *I*^2^ = 0.0%, (*p* <0.0001)	1.46	(1.25, 1.72)	100.00
**≥1-year mortality**			
Patel (15)	4.97	(3.06, 8.09)	12.47
Pizzonia (6)	5.63	(2.31, 13.75)	7.09
Winter (19)	3.50	(2.10, 5.70)	12.25
Jorissen (21)	1.25	(1.11, 1.41)	17.84
Thorne (23)	2.87	(2.10, 3.92)	15.36
Choi (20)	1.42	(1.24, 1.63)	17.69
Choi (22)	1.49	(1.25, 1.77)	17.30
Total (95% CI) *I*^2^ = 91.3%, (*p* <0.0001)	2.24	(1.66, 3.04)	100.00
**Adverse discharge destination**			
Thorne (23)	0.8	(0.46, 1.39)	54.40
Chan (17)	23.00	(3.00, 173.50)	45.60
Total (95% CI) *I*^2^ = 89.8%, (*p* > 0.05)	3.70	(0.14, 98.22)	100.00
**Prolonged hospital stays**			
Choi (20)	1.19	(1.05, 1.34)	80.82
Thorne (23)	1.02	(0.80, 1.31)	19.18
Total (95% CI) *I*^2^ = 14.8%, (*P* = 0.01)	1.15	(1.03, 1.28)	100.00

#### Complications

Four studies were included to examine the predictive value of frailty on postoperative or in-patient complications. The heterogeneity test suggested that *I*^2^ = 77.4%, so a random-effects model was applied for the meta-analysis. The results demonstrated that frail elders were more likely to experience complications after hip fracture (OR, 1.46; 95% CI, 1.13–1.90). There were two studies from the same research team in the included studies. According to this, we conducted a subgroup analysis and found heterogeneity disappeared in the case of grouping (Figure 3 in the Supplementary File).

**Figure 3:**
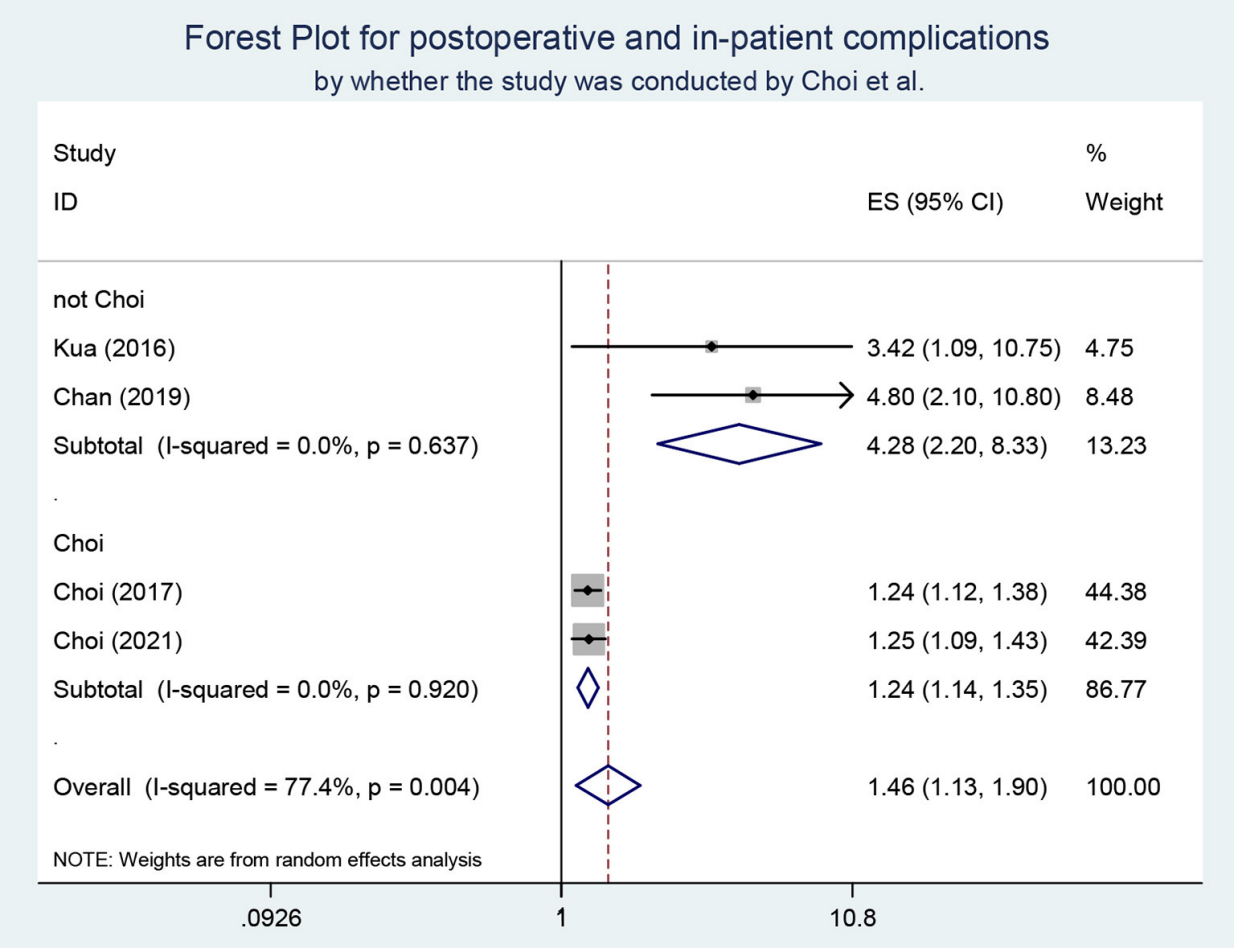
Subgroup analysis for the studies reporting post-operative and in-patient complications.

#### Other Adverse Outcomes

There were 2 articles included exploring the impact of frailty on discharge destination. The results prompted that there was no obvious difference between frail and fit elders in terms of adverse discharge destination (*p* > 0.05). It is necessary to acknowledge that the small number of included studies might affect the accuracy of the result. Our study also explored the fact that frail individuals were more likely to spend prolonged hospitalization (OR, 1.15; 95% CI, 1.03–1.28; *I*^2^ = 14.8%). However, the different definitions of prolonged hospital stays may impact the results of our analysis.

## Discussion

The concept of frailty was first introduced by the Federal Conference on Aging in the United States and usually refers to the dynamic process of declining multisystem function (25). There are various methods for frailty evaluation, but the uniform standard has not been identified. Among the articles we included, various assessment tools derived from Frailty Index, Frailty Phenotype, and Clinical Frailty Scale were mainly included. Most focused on several aspects such as endurance, fatigue, mobility, and disease. Hip fracture is a relatively serious type of fracture and can be even the last fracture of an individual's life. A possible reason for this is the increased risk of infection (Respiratory and urinary systems) and reduced cardiac function associated with prolonged bed rest (26).

Now-a-days, more researchers have focused on frailty screening in clinical practice. In a meta-analysis of 2,278 participants, the researchers evaluated that the frailty phenotype could be a valuable risk assessment tool for preoperative screening (27). Similarly, the results of our meta-analysis were consistent with the previous study, indicating that frailty evaluation should be applied to aged patients. Our study reconfirmed the predictive role of frailty on poor outcomes after a fracture. Hence, frailty can be considered as an intermediate step from self-care to disability. Early identification of frailty and prevention of frail elders toward disability will play an immensely prominent role in healthy aging.

In our meta-analysis, frail older were at a higher risk to suffer complications and prolonged hospital stays. In the study of the association between frailty and postoperative complications, *I*^2^ was 77.4% and we found that *I*^2^ was 0.0% in the respective subgroups. We considered that the reason was probably partly since the frailty assessment tool in Choi's study was Hip-MFS and the adjusted OR referred to the increased risk along with per 1 point increase in Hip-MFS. The hip fracture could be strong stress for elders. Weak physical conditions were more likely to lose stability under the impact of stress (13). In addition, frailty was associated with both short-term mortality and long-term mortality. In the study of the relationship between frailty and long-term mortality, we did subgroup analyses based on the region of the experiment and the type of study design. The results showed that frailty was a stronger predictor of mortality in the European and American studies and prospective studies. Although frail elders had no significant risk higher in adverse discharge destination in our research results. The study of Chan et al. had shown that seniors with higher frail scores were more likely to experience an adverse discharge destination (discharge to long-term care or death) (17). However, there was no significant difference in Thorne's study. One of the reasons may be that patients who previously lived in a nursing home and death were not included (23). Moreover, the number of studies and sample size were both small, and more research was required in the future to explore the predictive value of frailty on discharge destination.

Previous meta-analyses have demonstrated the relationship between frailty and surgical outcomes (27–29), but there are fewer pooled analyses on the specific population of hip fracture patients. Therefore, our study is dedicated to enriching this field of research. In our research, we have discovered that frailty might be a good predictor of adverse outcomes in aged hip fracture patients. Frail seniors are more vulnerable to suffer undesirable complications, increased mortality, and prolonged hospitalization. There are several limitations in our study which should be taken into consideration. First, not all articles are at a high level of quality. Low-quality (<7 scores) studies are also included in the analysis to access the results of larger samples and more studies. There may be comparability issues with the included studies. One of the conditions for evaluating cohort studies as comparable can be understood as whether the experiment is corrected for important confounding factors. We evaluated all articles, but inevitably, the poor comparability of the included articles leads to compromised veracity of the merged effects worth. Confounding factors in the article can have an impact on the study results. Therefore, we combined the adjusted ORs and confidence intervals for the analysis whenever possible, which would reduce the effect of confounding factors to some extent. Second, the measures of frailty assessment in the included 11 studies are inconsistent, contributing to the obvious heterogeneity across our studies. Third, only 2 studies are involved to explore the impact of frailty on discharge destination and prolonged hospitalization, which may affect the reliability of the conclusions. Additionally, the differences in study design might be a source of heterogeneity. Frailty is a non-specific state in which the organism is less responsive to internal and external environmental stressors. The gold standard on the assessment of debilitation has not yet been established. Differences in frailty assessment tools do account for a significant amount of heterogeneity, but we believe the results of this study are still informative. At last, prospective studies have stronger arguments and higher reliability than retrospective studies. However, the included 11 articles only involve 4 prospective studies.

Our study is not only intended to highlight the relationship between frailty and poor outcomes, but also to raise awareness of the importance of early identification and prevention of frailty. The prevalence of frailty in elders is currently high and is anticipated to increase further as global aging accelerates. According to statistics, the incidence of frailty among community-dwelling seniors fluctuated from 14.9 to 31.9%, which will vary with assessment tools (30). The prevalence of frailty in hospitalized geriatric patients is higher (31). A study data has shown that the likelihood of falling is 1.8 times higher for the frail than for the healthy elders (5). Concerning the strengths and implications of the study, our study suggests a significant correlation between preoperative frailty status and poor prognosis in aged hip fracture patients. Frailty seems to be more reflective of the physical condition of older individuals compared to age. The application of frailty assessment to geriatric patients is something that should be widely implemented in future clinical practice, and caring for frail geriatric patients and providing some early interventions may reverse the occurrence of poor outcomes. Current interventions for frailty include the following: exercise, nutritional supplementation, enhanced treatment of existing conditions, and medical injury reduction. We propose that there is a strong causal relationship between the events of frailty, falls, fractures, and the occurrence of postoperative adverse outcomes. Focusing on the frailty status of older adults will be important to promote healthy aging.

In summary, frailty, fall, fractures, and the increased risk of complications and mortality combine to constitute a series of causal associations that will have a serious threat to the health of older adults. In future medical practice, frailty has the potential to be an effective tool for risk stratification of aged patients and thus guide disease prevention and treatment.

## Conclusion

This meta-analysis was conducted mainly to explore the impact of frailty on poor outcomes in hip fracture patients. We conclude that frailty is a good predictor for adverse complications, increased mortality, and prolonged hospitalization.

## Data Availability Statement

The original contributions presented in the study are included in the article/Supplementary Material, further inquiries can be directed to the corresponding author.

## Author Contributions

YS and ZW retrieved the literature and independently evaluated the potentially eligible studies. They respectively extracted relevant data from the included articles and conducted the meta-analysis. HH provided assistance in the data analysis process. When disagreements arose, they discussed and consulted with the corresponding author (PZ) to reach a consensus. YS wrote this manuscript under the supervision and assistance of PZ. All authors contributed to the article and approved the submitted version.

## Conflict of Interest

The authors declare that the research was conducted in the absence of any commercial or financial relationships that could be construed as a potential conflict of interest.

## Publisher's Note

All claims expressed in this article are solely those of the authors and do not necessarily represent those of their affiliated organizations, or those of the publisher, the editors and the reviewers. Any product that may be evaluated in this article, or claim that may be made by its manufacturer, is not guaranteed or endorsed by the publisher.
