# Supplementary material for: The Impact of Frailty on Adverse Outcomes in Geriatric Hip Fracture Patients: A Systematic Review and Meta-Analysis
Source: Front Public Health. 2022 Jun 30;10:890652. doi: 10.3389/fpubh.2022.890652 (PMC9280195; doi:10.3389/fpubh.2022.890652)
Supplement: Supplementary file 1 [file Data_Sheet_1.docx]

## Forest plots

### Postoperative and in-patient complications

### Inpatient mortality

### 6-month mortality

### ≥1-year mortality

#### Subgroup analysis (1)

### Prolonged hospitalization

### Adverse discharge destination
