# Supplementary material for: The Impact of Frailty on Adverse Outcomes in Geriatric Hip Fracture Patients: A Systematic Review and Meta-Analysis
Source: Front Public Health. 2022 Jun 30;10:890652. doi: 10.3389/fpubh.2022.890652 (PMC9280195; doi:10.3389/fpubh.2022.890652)
Supplement: Supplementary file 2 [file Table_1.pdf]

Search strategy for PubMed (404)

ID	Search
#1	(Frail[Title/Abstract]) OR (frailty[Title/Abstract])
#2	hip fractures[MeSH Terms]
#3	((((Femoral Fracture*[Title/Abstract]) OR (Intertrochanteric Fracture*[Title/Abstract])) OR (Trochanteric Fracture*[Title/Abstract])) OR (Femoral Neck Fracture*[Title/Abstract])) OR (Subtrochanteric Fracture*[Title/Abstract])
#4	#2 OR #3
#5	#1 AND #4
#6	#5 Publication Year from 1990 to 2021
#7	#6 excluded review

Search strategy for Web of science (924)

ID	Search
#1	(TS=(frail)) OR TS=(frailty) OR TS=(frail elderly)
#2	(((((TS=(hip fracture*)) OR TS=(Femoral Fracture*)) OR TS=(Femoral Neck Fracture*)) OR TS=(Intertrochanteric Fracture*)) OR TS=(Trochanteric Fracture*)) OR TS=(Subtrochanteric Fracture*)
#3	#1 AND #2
#4	#3 Publication Date 1990-01-01 to 2021-10-10
#5	#4 excluded review articles, Meeting, Patent, Editorial materials, letters, case report;

Search strategy for Cochorane of library (161)

ID	Search
#1	(frail):ti,ab,kw OR (frailty):ti,ab,kw OR (frail elderly):ti,ab,kw
#2	hip fractures
#3	(hip fracture*):ti,ab,kw OR (Femoral Fracture*):ti,ab,kw OR (Femoral Neck Fracture*):ti,ab,kw OR (Intertrochanteric Fracture*):ti,ab,kw OR ((Trochanteric Fracture*) OR (Subtrochanteric Fracture*)):ti,ab,kw
#4	#2 OR #3
#5	#1 AND #4

#6	#5 limit 1990-01 to 2021-10
#7	**Limit to trails**

Search strategy for Embase(299)

ID	Search
#1	'frail elderly'/exp OR 'frailty'/exp OR 'frail'
#2	'hip fracture'/exp
#3	'hip fracture*':ab,ti OR 'femoral fracture*':ab,ti OR 'femoral neck fracture*':ab,ti OR 'intertrochanteric fracture*':ab,ti OR 'trochanteric fracture*':ab,ti OR 'subtrochanteric fracture*':ab,ti
#4	#2 OR #3
#5	#1 AND #4
#6	#5 limit 1990-01 to 2021-10
#7	#6 AND 'article'/it<br>AND ('clinical article'/de OR 'cohort analysis'/de OR 'observational study'/de OR 'prospective study'/de OR 'retrospective study'/de)
