# Supplementary material for: The Impact of Frailty on Adverse Outcomes in Geriatric Hip Fracture Patients: A Systematic Review and Meta-Analysis
Source: Front Public Health. 2022 Jun 30;10:890652. doi: 10.3389/fpubh.2022.890652 (PMC9280195; doi:10.3389/fpubh.2022.890652)
Supplement: Supplementary file 3 [file Table_2.pdf]

Supplementary file2

Newcastle-Ottawa Scale for assessing the quality of studies in meta-analysis

Study	Selection					outcome			scores
	Ascertainment of exposure	Representativeness of the exposed cohort	Selection of the nonexposed cohort	outcome of interest was not present at start of study	Comparability	Assessment of outcome	Was follow-up long enough for outcomes to occur	Adequacy of follow up of cohorts	
Patel et al, 2014	1	1	1	1	0	1	1	1	7
Kua et al, 2016	1	1	1	1	0	0	1	1	6
Chan et al, 2019	1	1	1	1	0	1	1	1	7
Chen et al, 2019	1	1	1	1	1	1	1	1	8
Winters et al, 2018	1	1	1	1	0	1	1	1	7
Choi et al, 2017	1	1	1	1	0	0	1	1	6
Jorissen et al, 2020	1	1	1	1	0	1	1	1	7
Choi et al, 2021	1	1	1	1	0	1	1	1	7
Thorne et al, 2021	1	1	1	1	0	0	1	1	6
Pizzonia et al, 2021	1	1	1	1	0	1	1	1	7
Shimizu et al., 2021	1	1	1	1	0	1	1	1	7
